# Outcomes of Laparoscopic Treatment Modalities for Unilateral Non-palpable Testes

**DOI:** 10.3389/fped.2016.00013

**Published:** 2016-03-04

**Authors:** Nurullah Hamidi, Onur Telli, Uygar Bagci, Baris Esen, Mehmet Ali Karagoz, Ahmet Metin Hascicek, Tarkan Soygur, Berk Burgu

**Affiliations:** ^1^Department of Urology, Ankara University School of Medicine, Ankara, Turkey; ^2^Department of Pediatric Urology, Ankara University School of Medicine, Ankara, Turkey; ^3^Department of Urology, Ankara Training and Research Hospital, Ankara, Turkey

**Keywords:** Clavien, complication, laparoscopy, non-palpable testis, Fowler–Stephens orchiopexy

## Abstract

**Purpose:**

To date, laparoscopy has gradually become the gold standard for treatment of non-palpable testicles (NPT) with different success and complication rates. In this study, we aimed to evaluate outcomes of laparoscopic approaches for NPT.

**Materials and Methods:**

We reviewed data of 82 consecutive patients who underwent laparoscopic treatment for unilateral NPT at two institutions by two high volume surgeons from 2004 January to 2014 December. Laparoscopic-assisted orchidopexy (LAO) and two-stage Fowler–Stephens technique (FST) was performed for 45 and 37 patients, respectively. Age (at surgery), follow-up time, laterality of testes, and postoperative complications were analyzed. Modified Clavien classification system (MCCS) was used for evaluating complications.

**Results:**

The median age (at surgery) and median follow-up time were 18 (range: 6–56) and 60 (range: 9–130) months, respectively. Overall success rate for two laparoscopy techniques was 87.8% during the maximal follow-up time. We observed wound infection in two, hematoma in one, testicular atrophy in five, testicular re-ascending in two patients at follow-up period. There was no statistical difference between two laparoscopic techniques for grade I (five vs. two patients, *p* = 0.14) and grade IIIb MCCS complications (five vs. two patients, *p* = 0.44).

**Conclusion:**

Our results have shown that two laparoscopic approaches have low complication rates.

## Introduction

Undescended testis (UT) is the most common congenital anomaly of genitalia in newborn male infants. It occurs in approximately 3% of term and 30% of preterm infants (1). The etiology is still unclear, despite its frequency. Some factors are effective in occurrence of UT such as low birth weight, short gestational period, low intra-abdominal pressure, chromosomal anomalies, and hormonal factors (2, 3). Early diagnosis and treatment of UT is important because of fertility improvement and adequate physical examination (for increased risk of testicular malignancy) (4).

According to clinical assessment, UT divided into two groups: palpable and non-palpable. Approximately 20% of UT cases are non-palpable and non-palpable testicles (NPT) may be intra-abdominal, canalicular, atrophic, or absent. There is no superiority of radiological imaging modalities over physical examination for evaluation of NPT. In 1976, since Cortesi et al. described the use of laparoscopy for NPT evaluation, laparoscopy has gradually become the gold standard for localization (with 99% sensitivity) and eventual treatment of NPT when the testicle is still non-palpable under general anesthesia (5).

In this study, we aimed to evaluate our laparoscopic treatment outcomes and postoperative complications with modified Clavien classification system (MCCS) over 10-year period.

## Materials and Methods

The study was approved by the Research Ethics Committee of Ankara University School of Medicine. We assessed data of 151 unilateral NPT who underwent laparoscopy at two institutions by two high volume surgeons, between January 2004 and December 2014. Data of patients were analyzed and included age (at surgery), follow-up time (month), laterality of testes, and postoperative complications. Postoperative complications (wound infection, hematoma, atrophy, and testicular re-ascending) were evaluated with MCCS. Wound infection and hematoma were considered at early postoperative period. Postoperative testicle size was measured with prader orchidometer according to contralateral testicle size at every visit till final visit. Testicular re-ascending was considered when testicles located above the scrotum at every visit till final visit.

### Surgical Technique and Choice of Laparoscopic Approach

Prior laparoscopy, physical reexamination was performed for confirmation of NPT diagnosis by surgeons after general anesthesia induction. Patients who have become palpable after general anesthesia induction were excluded from study. For all patients, laparoscopic examination was performed at same position (mild Trendelenburg). After Foley catheter placement, open Hanson technique was chosen for subumbilical trocar (5 mm) placement. Intra-peritoneal pressure was kept in 8–10 mm Hg. Vas deferens and spermatic vessels were evaluated in the only affected side. If required, two additional trocars were used.

In 17 (11.2%) patients, blind-ending spermatic vessels were observed at the time of surgery, and they were considered as complete testicular atrophy (vanishing testis). Testicular absent was considered (nine cases) if patients have no spermatic vessels and vas deferens during laparoscopic examination. In 22 (14.6%) testes, testicular vessels and vas deferens were seen entering in internal inguinal ring, inguinal exploration was performed. In 14 (9.3%) of them, we found viable testicles (canalicular testicles), and we performed inguinal orchidopexy during the same procedure. However, nubbin tissue was observed in eight (5.3%) patients, and we performed nubbinectomy. On histopathological confirmation of nubbinectomy, germ cell was not identified in all of patients. In 23 (15.2%) testes, atrophic testicles were observed via diagnostic laparoscopy, and laparoscopic orchiectomy was performed because of high risk of testicular cancer development. On histopathological examination, severe atrophic and fibrotic changes were resulted in all orchiectomy specimens. Cases of testicular absent, atrophic, canalicular testis, and nubbin tissue were excluded from study (totally 69 testes).

**Figure 1:**
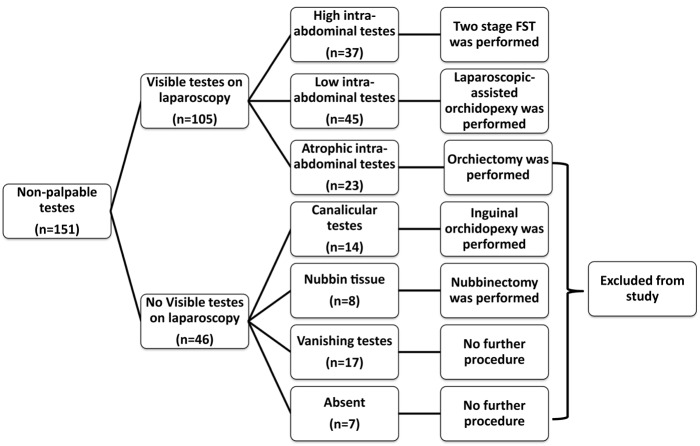
**Flow chart of current study**.

Among the remaining 82 testes, we performed laparoscopic-assisted orchidopexy (LAO) or two-stage Fowler–Stephens technique (FST) orchidopexy for 37 (24.5%) and 45 (29.8%) testicles, respectively. Flow chart of current study was demonstrated in Figure 1. LAO was performed if testis is at low position (<2 cm diameter, between testis and internal inguinal ring) and if testis can move easily to around contralateral internal inguinal ring after spermatic vessels dissection. Two-stage FST performed if testis is at high position (>2 cm diameter, between testis and internal inguinal ring). In cases of two-stage FST, the second step was performed 6 months after first stage if presence of adequate testicle size.

### Statistical Analysis

All statistical analyses were performed with SPSS ver.16.5 (Statistical Package for Social Sciences for Windows 16.5 Inc., Chicago, IL, USA). Age and follow-up time were assessed with non-parametric test, and all values were expressed as median values with range. Comparison of complications between two groups was evaluated with Chi-square test. *p*-value less than 0.05 was considered to indicate statistical significance.

## Results

The UTs were right sided in 61% (92 testicles), left sided in 39% (59 testicles) of all cases. The median age (at surgery) and median follow-up time of all patients were 18 (range: 6–56) and 60 (range: 9–130) months, respectively. There was no statistical difference in median follow-up time (61 and 58 months, *p* > 0.05) and median age (18 and 17 months, *p* > 0.05), between two laparoscopic approaches.

We did not observe intra-operative complications. At follow-up after surgery, wound infection was observed in one patient for each group on trocar placement line in few days after surgery. Hematoma was not observed in patients who performed two-stage FST, but it was observed only in one patient after LAO. Testicular re-ascending was observed in only two (5.4%) cases in two-stage FST (after second step) groups. It was observed at 3 months after surgery in one of these patients and at 6 months after surgery in other patients. These patients re-operated because of this reason. There was no case of testicular re-ascending in LAO technique. The most common complication (50% of all complications) was testicular atrophy, and it was observed in one patient in LAO groups at 1 month after surgery. In FST group, testicular atrophy was observed in four patients after first step. Therefore, we performed orchiectomy for these patients during second step. We did not observe testicular atrophy after second step.

The postoperative complications were classified with MCCS (6). According to MCCS, wound infection and hematoma were considered as grade I, testicular atrophy and re-ascending were considered as grade IIIb because of requiring further surgical procedure under general anesthesia. However, in two-stage FST group, testicular atrophy was not considered as grade IIIb complication. Because, we observed atrophy and performed orchiectomy at the second step of FST. Therefore, we did not need to perform additional surgery under general anesthesia. So, grade I complications were observed in five (13.5%) and two (4.4%) patients, for LAO and two-stage FST approaches, respectively, and there was no statistically significant difference for MCCS grade I complications between two laparoscopic techniques (*p* = 0.14). Grade IIIb complications were observed in one (2.2%) and two (5.4%) patients, in LAO and two-stage FST groups, respectively and this difference was not statistically significant (*p* = 0.44). All complications were detailed in Table 1.

**Table 1:** **Comparison of complications between two laparoscopic procedures**.

Grade of complication according to MCCS	Two-stage FST (37 patients) *n*, %	Laparoscopic-assisted orchidopexy (45 patients) *n*, %	*p* value
**Grade I**			
Wound infection	1, 2.7%	1, 2.2%	
Hematoma	–	1, 2.2%	
Atrophy	4, 10.8%	–	
Totally	5, 13.5%	2, 4.4%	0.14
**Grade IIIB**			
Atrophy	–	1, 2.2%	
Testicular re-ascending	2, 5.4%	–	
Totally	2, 5.4%	1, 2.2%	0.44

*MCCS, Modified Clavien Classification System; FST, Fowler–Stephens technique*.

## Discussion

In 1929, Cooper et al. reported the first article about presence of histological changes in UT cases. They defined that histological changes were observed after 18 months of age in children who have UT (7). Furthermore, Hadziselimovic et al. have been reported about importance of early orchidopexy for improvement germ cell maturations and fertility rate, in their articles (8, 9). Then, Canavese et al. reported their 12-year experience in 916 children (10). They emphasized that orchidopexy must be performed till 12 months of age.

About one-fifth of UT cases are non-palpable on physical examination (4). Previously, ultrasonography, computed tomography, magnetic resonance imaging, gonadal arteriography, and venography have been tested for localization of NPT (11–15). Nowadays diagnostic laparoscopy widely accepted for NPT evaluation despite many non-invasive radiological investigations. First, the importance of laparoscopy on NPT evaluation was described by Cortesi et al. (5). Then, studies have gradually widespread, and diagnostic accuracy of laparoscopy on NPT diagnosis was demonstrated by many authors (16, 17).

More recently, many authors have published their cumulative experience about different laparoscopy techniques for NPT management such as LAO, one or two-stage FST (4, 18–21). Esposito et al. reported their complication rates of LAO and two-stage FST techniques for NPT cases (LAO in 24 cases, two-stage FST in only one case) during 3-year period (18). They evaluated testis size and viability with power color doppler ultrasonography. Intra-operative complication (spermatic vessels damage) was observed in only one (4%) patient who underwent two-stage FST. In LAO group, postoperative testis size was smaller than contralateral testis despite normal vascularization on power color doppler examination in five (20%) patients. In another study from Egypt, safety and efficacy of laparoscopic approaches were evaluated with postoperative atrophy and viability (via Technetium-99 m) and testicular re-ascent (4). According to this study, in LAO group (46 patients) postoperative atrophy, viability, and testicular re-ascending rates were 0, 100, and 8.7%, respectively. In two-stage FST group (47 patients), these rates were 4.3, 95.7, and 0%, respectively. The authors were emphasized that success of laparoscopy depends on selection of laparoscopic surgery technique according to distance between testis and internal inguinal ring. Denes et al. reported the success rates in totally 54 testes (two-stage FST in 25 and primary laparoscopic orchidopexy in 29 testicles) (19). In this study, primary laparoscopic orchidopexy was performed in two techniques: with and without vascular ligature (3 and 26 testicles, respectively). Their reported 88, 33, and 96% success rates for two-stage FST, primary laparoscopic orchidopexy with vascular ligature and without vascular ligature, respectively. They commented that surgeon should be aware when performed primary laparoscopic orchidopexy with vascular ligature. Lotan et al. published an article about success rate of LAO (in 12 patients) and two-stage FST (in 54 patients) (20). In their study, postoperative atrophy was observed only in two patients (3.4%) after two-stage FST orchidopexy. In a large multi-­institutional study, Baker at al. evaluated complications (atrophy and testis retraction) for three laparoscopic orchidopexy approaches (primary laparoscopic orchidopexy, one-stage FST, and two-stage FST) in 263 testicles (21). In this study, atrophy occurred in 2.2, 22.2, and 10.3% of patients who underwent primary laparoscopic orchidopexy, one-stage FST and two-stage FST, respectively. On follow-up, testicular retraction was observed 0.6, 7.4, and 1.7% of testicles in three groups, respectively.

In present study, we report complication rates of two laparoscopic procedures in 82 patients over a 10-year period (median follow-up time: 60 months). Intra-operative complication was not observed in our patients. Our postoperative wound infection, hematoma, atrophy, and re-ascending rates are 2.2, 2.2, 2.2, and 0% for LAO technique and 2.7, 0, 10.8, and 5.4% for two-stage FST technique. These rates were compatible with the current literature. In FST group, we did not observe atrophy and re-ascending after second step. According to previous studies, our atrophy rates of FST group (after completion of second step) were lower. The reason may be due to lower patient age (median 18 months). Furthermore, the difference of this study from previous studies, the severity of complication was categorized based on MCCS. First, Clavien et al. described a classification for complications (22). Then, Dindo et al. modified it (6). This modification was performed to add further precision and to characterize whether an intervention due to the complication led to general anesthesia, intensive care unit admission, or organ failure, and again, it was based on the type of therapy required to treat the complication. In our patients, the most common complication was testicular atrophy, and it was observed in five (50% of all complicated cases) patients. The other complications (wound infection, hematoma and testicular retraction) were observed in two (20%), one (10%) and two (20%) patients, respectively. There was no statistically significant difference for MCCS grade I (13.5 vs. 4.4%, *p* = 0.14) and grade IIIb (5.4 vs. 2.2%, *p* = 0.44) complications between two laparoscopic techniques. Life-threatening complications such as Grade IV or V were not seen in our patients.

Our study has several limitations. This study is in retrospective nature and the number of patients decreased to 82, because of excluding data (data of vanishing, atrophic, canalicular testicle, and nubbin patients) from study. Particularly, we observed canalicular testis in 14 (9.2%) patients during laparoscopic exploration. Probably, these patients were either insufficiently worked up or not well examined even under general anesthesia. Maybe we should have evaluated these patients with radiological investigation such as ultrasonography for excluding of canalicular testis. In addition, the postoperative atrophy could not be evaluated objectively because of intra-operative testicle size may measure different than real testis size under laparoscopic magnification. Furthermore, we did not use any radiological imaging method for evaluation of postoperative testis size and testis viability. Maybe, we could use ultrasonography or power color Doppler investigation for more objective assessment of postoperative size and viability of testes.

## Conclusion

Laparoscopic exploration must be done in all of NPT cases for confirmation of diagnosis. Our results have shown that two laparoscopic approaches have low complication rates.

## Author Contributions

NH: protocol/project development, data collection, and manuscript writing, OT: data collection or management and manuscript writing, UB: data collection, BE: manuscript writing/editing/revising, AH: data collection, manuscript writing/editing, MK: data collection and manuscript writing/editing, TS: manuscript writing/editing, BB: project development and manuscript writing/editing.

## Conflict of Interest Statement

The authors declare that the research was conducted in the absence of any commercial or financial relationships that could be construed as a potential conflict of interest.
